# Body Fat Percentage and Long-Term Risk of Fractures. The EPIC-Norfolk Prospective Population Cohort Study

**DOI:** 10.1016/j.maturitas.2022.11.005

**Published:** 2022-12-02

**Authors:** Tiberiu A Pana, Sheng Hui Kioh, Samuel R Neal, Maw Pin Tan, Sumaiyah Mat, Alireza Moayyeri, Robert N Luben, Nicholas J Wareham, Kay-Tee Khaw, Phyo K Myint

**Affiliations:** 1 Ageing Clinical & Experimental Research Group, Institute of Applied Health Sciences, University of Aberdeen, Aberdeen, Scotland; 2 Ageing and Age-Associated Disorders Research Group, University of Malaya, Kuala Lumpur, Malaysia; 3 Department of Medicine, Faculty of Medicine, University of Malaya, Kuala Lumpur, Malaysia; 4 Department of Medical Sciences, School of Healthcare and Medical Sciences, Sunway University, Malaysia; 5 Center of Healthy Ageing and Wellness, Faculty of Health Sciences, National University of Malaysia; 6 University College London, London, United Kingdom; 7 Gonville and Caius College, University of Cambridge, United Kingdom; 8 MRC Epidemiology Unit, Institute of Metabolic Science, University of Cambridge, United Kingdom

**Keywords:** Ageing, Fracture, Body Composition, Osteoporosis

## Abstract

**Background:**

This cohort study aimed to determine the association between body fat percentage (BF%), incident fractures and calcaneal broadband ultrasound attenuation (BUA).

**Methods:**

Participants were drawn from EPIC-Norfolk (median follow-up = 16.4 years). Cox models analysed the relationship between BF% and incident all and hip fractures. Linear and restricted cubic spline (RCS) regressions modelled the relationship between BF% and BUA.

**Results:**

14,129 participants (56.2% women) were included. There were 1283 and 537 incident all and hip fractures respectively. Mean (standard deviation) age of 61.5 (9.0) years for women and 62.9 (9.0) years for men. Amongst men, BF% was not associated with incident all fractures. While BF%<23% (median) was not associated with hip fractures, BF%>23% was associated with increased risk of hip fractures by up to 50% (hazard ratio (95% confidence interval) = 1.49 (1.06-2.12)). In women, BF%<39% (median) was associated with up to 32% higher risk of all fractures (1.32 (1.13-1.44)), while BF%>35% was not associated with this outcome. Higher BF% was associated with lower risk of incident hip fractures in women. Higher BF% was associated with higher BUA amongst women. Higher BF% up to ~23% was associated with higher BUA amongst men.

**Conclusions:**

Higher BF% is associated with lower risk of fractures in women. While there was no association between BF% and all fractures in men, increasing BF% >23% was associated with higher risk of hip fractures in men. This appears to be independent of estimated bone mineral density. Fracture prevention efforts need to consider wider physical, clinical, and environmental factors.

## Introduction

The prevalence of osteoporotic fractures specifically hip fractures is expected to increase from 1.26 million in 1990 to 4.5 million in 2050 ^1^. Bony fractures in older adults also lead to increased functional dependence, reduced life expectancy and increased institutionalization ^2,3^. One in three older adults are no longer alive one year after a hip fracture, with survivors experiencing functional decline, disability and reduced quality of life. As a result, fractures in older adults lead to increased healthcare burden ^4^.

In addition to reduced bone mass leading to increased risk of osteoporotic fractures, ageing is also associated with sarcopenia and increasing fat mass. Sarcopenia is associated with mobility limitations, disability, falls and consequent fractures. The relationship between body fat and fractures is, however, less clear. It has been previously found that increased body fat is associated with reduced risk of all fractures but only in older women, but not in men ^5,6^. Nevertheless, there are also suggestions that body fat mass may increase fracture risk in older adults, specifically vertebral fractures^7^.

A previously published study evaluating the association between body fat and hospitalization due to fractures over a follow-up of 8.7 years using data from the European Prospective Investigation into Cancer (EPIC)-Norfolk data has identified gender differences in this association, with increased body fat demonstrating a potentially protective effect in women but not in men ^5^. However, the above study was limited by the lower number incident fractures at the time of follow-up. In this study, we aimed to identify the relationship between body fat mass and incident hip and all fracture hospitalisations as well as calcaneal broadband ultrasound attenuation (BUA) with an additional 8 years of follow-up since the previous analysis.

## Methods

### Study Participants

Participants were drawn from the EPIC-Norfolk prospective population study. Details of the design, study procedures and participant recruitment have been published previously ^8^. Briefly, the cohort recruited adults aged 40 – 79 years old between 1993 – 1997 from age-sex registers of 35 participating general practices in Norfolk, United Kingdom (UK). A total of 25,639 participants attended the baseline health check and completed a detailed self-reported health and lifestyle questionnaire. This study was approved by the Norwich Local Research Ethics Committee and all participants have provided written consent prior to their participation. Participants from the baseline health check were invited to return for the second health check three years later (1998-2000) with the addition of percentage body fat using body-impedance analysis and bone mineral density using calcaneal ultrasound. A total of 15,027 participants completed the second follow-up measurements. Data from the second health check are included in this analysis. After the exclusion of participants with missing data on key variables, a total 14,129 men and women were included in the analyses.

### Data Collection

Data collection comprised of a detailed self-reported questionnaire on participant’s health and lifestyle as well as a clinic visit which include anthropometric measurements, body composition measurement and broadband ultrasound attenuation (BUA). Participant’s smoking status was derived from the following questions: “Are you currently smoking?” and “Have you ever smoked before?” while alcohol consumption was derived from the question “How many alcohol units you drink per week?”. Data regarding menopausal status and hormone replacement therapy were derived from the following questions from the health and lifestyle questionnaire administered at the second health check “If you are still menstruating, do you take hormones for the treatment of menopause?”, “If you are not still menstruating, how old were you when you stopped having your periods?”, “If you have ever received any hormone replacement therapy, are you currently taking this treatment?”, “How old were you when you started this hormone replacement therapy (HRT)?”, “Have you ever had a hysterectomy (womb removed)?”. Additionally, self-reported medical condition including myocardial infarction, diabetes mellitus, cancer, stroke and asthma and information on medication intake including antidepressants, antihypertensives, statins and aspirins were also obtained.

#### Testosterone Levels

Testosterone levels were quantified as part of a previous study from frozen serum samples taken from study participants at the baseline visit^9^.

#### Anthropometric Measurements

Height, measured using a free-standing stadiometer, and weight, measured using digital scales, were obtained by trained nurses on participants with their light clothing and without shoes. Body Mass Index (BMI) was calculated from the height in metres and weight in kilograms using the relationship: weight/height^2^. Waist and hip circumference were obtained using a D-loop non-stretch fibreglass tape placed between the ribs and the iliac crest and between the iliac crest and crotch of a standing participant respectively. Waist hip ratio (wasi) was then further calculated as waist circumference divided by hip circumference. All circumferences were measured to the nearest 0.1cm.

#### Body Composition

A bio-electric impedance analysis (BIA) device (Biostat™, Isle of Man, UK) was used to measure the total body water and fat free mass. Total body fat was calculated as body weight minus fat-free mass and converted to percentage body fat (%BF) accordingly based on their total body weight. Validation and reliability of this technique was discussed previously ^10,11^.

#### Broadband Ultrasound Attenuation

Calcaneal broadband ultrasound attenuation (BUA; db/MHz) and speed of sound (VOS; m/s) were obtained using the calcaneal ultrasound broadband attenuation (CUBA) sonometer (McCue Ultrasonics, Winchester, UK). At least two measurements of the left and right calcaneus were obtained, and the average measurement was taken in this analysis. Five CUBA machines, calibrated daily with a physical phantom, monthly with a roving platform and further compared on either the left or right calcaneus. This method has been discussed previously ^12,13^. Only the BUA was considered in this analysis owing to the high correlation between BUA and VOS.

#### Case Ascertainment

Mortality was ascertained using death certificate data from the Office of National Statistics. Participant unique identifiers were also linked to the National Health Service (NHS) hospital information system so that admission of NORFOLK residents anywhere in the UK was notified to EPIC-Norfolk. The dataset was also linked to ENCORE (East Norfolk Commission Record) for admission episodes. Incident fracture-related hospitalisations were ascertained using death certificate data or hospital discharge code with the International Classification of Diseases (ICD) 9 or ICD 10 codes for fractures by site obtained through record linkage with the National Health Service (NHS) hospital information system and ENCORE (East Norfolk Commission Record) to allow notification of any hospital admission (Supplementary Table 2). The follow up period of an average of 16 years per participant was defined as the time interval between the date of the second health check and the date of death for those who have died or the date of first fracture hospitalisation for those who had a hospitalization following a fracture and the end of follow-up (31^st^ March 2016) for the remaining participants.

### Data analysis

Stata SE 15.1 was used for statistical analyses. All descriptive and analytical statistics were performed separately for men and women. The χ^2^-test (for categorical data), one-way analysis of variance (for normally-distributed continuous data) and Kruskal-Wallis test (for non-normally-distributed continuous data) were used to compare patient characteristics between categories. Comparisons of baseline characteristics were conducted by categorising the study population according to BF% quartiles by gender.

The previous EPIC-Norfolk study suggested the association between BF% and our outcomes did not follow a linear trend ^5^. Hence, we used restricted cubic splines (RCSs) to model the relationship between BF% and each outcome as a flexible function. Goodness-of-fit statistics (Akaike and Bayesian Information Criteria) were calculated for the linear model and for RCS models with varying degrees of freedom (df=2 to df=7). The model with the lowest AIC was chosen for each outcome. Supplementary Table 1 details the best fitting models for each regression. The likelihood ratio test was used to confirm that the chosen model had a better fit than the linear model. After verification of the proportional hazards (PH) assumption, a Cox proportional hazards regression was modelled for each outcome. Cox regressions in which BF% was modelled using RCSs, the median BF% was chosen as reference (hazard ratio = 1). These models were adjusted for age, past history of fracture, height, smoking status, alcohol intake, physical activity, waist-hip ratio and BUA. Analyses including women were also adjusted by menopausal status and HRT use. Linear and RCS regression models were also created for the outcome of BUA, adjusted for the same covariates as before. In order to avoid over-fitting outliers, the analysis was only performed for values of BF% between the 5^th^ and 95^th^ percentiles for the respective population subgroup.

Using the same statistical methodology outlined above, subgroup analyses were also undertaken to explore the relationship between the hormonal environment and the associations outlined in the main analyses. Amongst the 2447 men included in the study for whom testosterone levels had been quantified based on frozen serum sampled at the first health check, further analyses evaluating the associations between body fat percentage and incident hip fractures were undertaken stratifying by testosterone levels (low < 25^th^ percentile versus high ≥ 25^th^ percentile). Similarly, further analyses of the relationship between body fat percentage and incident all and hip fractures were undertaken amongst women, stratifying by hormonal milieu: pre-menopausal women and those undergoing HRT versus post-menopausal women.

## Results

### Baseline characteristics and crude outcome rates

Data on body composition and fracture-related hospitalization were available for 14,129 participants (56.2% women) with mean (standard deviation) age of 61.5 (9.0) years for women and 62.9 (9.0) years for men. Median (inter-quartile range) follow-up or the entire cohort was 16.4 (15.7-17.1) years. Tables 1 and 2 detail the baseline characteristics of the included cohort, stratified BF% quartile for women and men respectively. Supplementary Tables 3 and 4 detail the baseline characteristics stratified by the median BF% for women and men respectively. Significant differences were observed across quartiles of BF% in women except for the mean age of menopause, previous history of fracture and incident all fractures. Amongst men, there were significant differences in terms of baseline characteristics between the BF% quartiles except for mean age, previous history of fractures and incident all and hip fractures.. Over the study follow-up, 399 (5.02%) women and 138 (2.2%) men experienced incident hip fractures, respectively, while 909 (11.4%) women and 374 (6.1%) men experienced any incident fracture, respectively.

### Body fat percentage and all incident fractures

**Figure 1:**
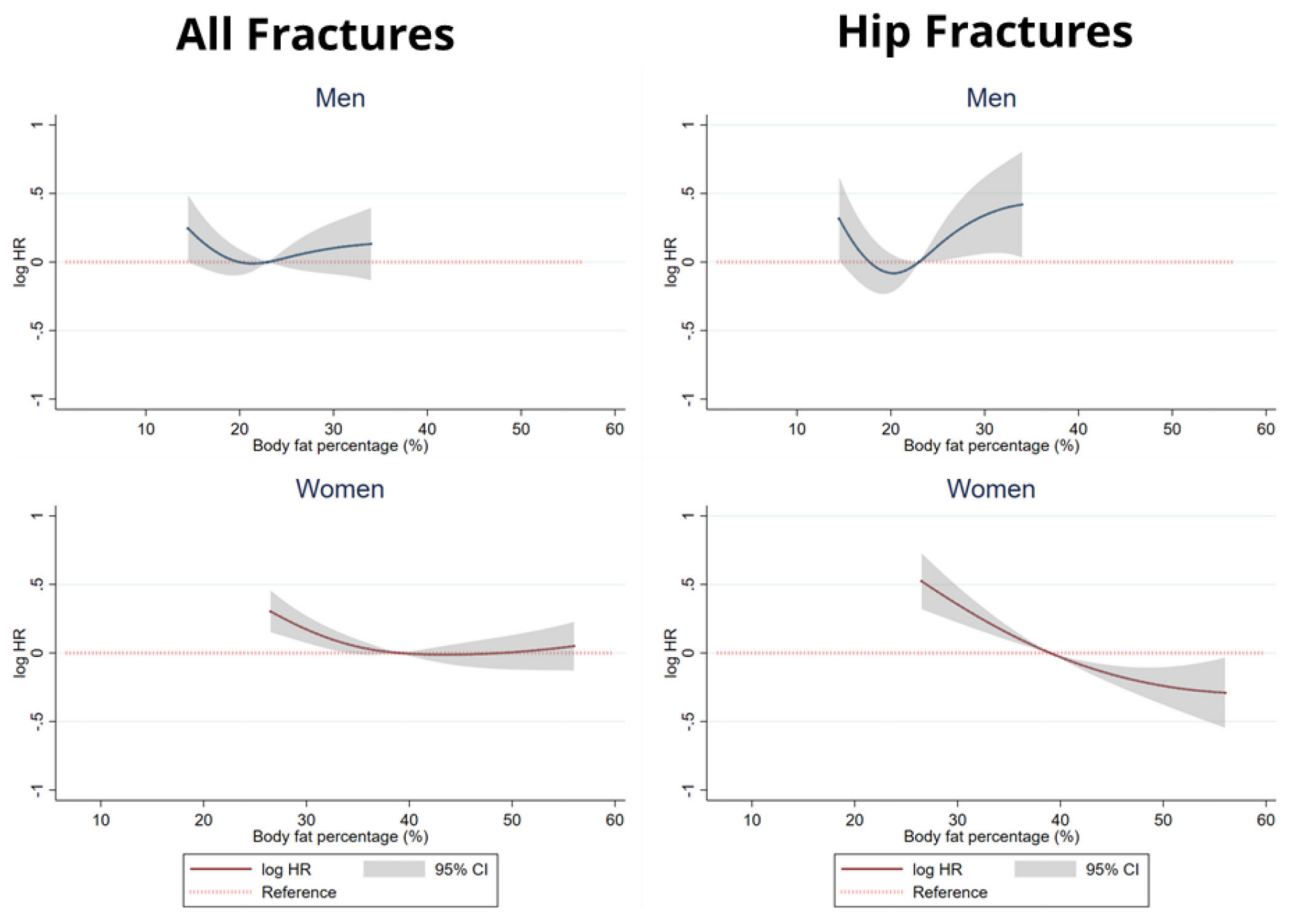
Results of multivariable Cox regressions assessing the association between body fat percentage (BF%) and incident all and hip fractures in 14,129 included men and women participants of the EPIC-Norfolk study over a median of 16.4 years of follow-up. Among men, the hazard ratios describing the relationship between BF% and incident fractures were modelled using a linear model and restricted cubic spline (RCS) model with 2 internal knots for both all and hip fractures. Among women, RCS models with 2 and 1 internal knot were used for incident all and hip fractures respectively. The natural logarithm of the hazard ratios and respective 95% confidence intervals are represented by the solid line and grey shadowing respectively. The dotted red line represents the reference line (log HR = 0 / HR = 1). Predicted hazard ratio values are displayed for BF% values ranging between the 5^th^ and 95^th^ percentiles of the analysed population. All models were adjusted for age, past history of fracture, height, smoking status, alcohol intake and broadband ultrasound attenuation. HR = hazard ratio; CI – confidence interval;

Figure 1 details the association between BF% and all incident fractures stratified by sex. Significant inverse associations were present between BF% and all incident fractures in women but not in men after full adjustment. Compared to women with the median BF% (39.0%), those with a BF% less than 39% were up to 32% more likely (hazard ratio (95% confidence interval) = 1.32 (1.13-1.44)) to suffer an incident fracture. Compared to the median BF%, there were no associations between BF% >39% and incident fractures.

### Body fat percentage and hip fractures

Figure 1 also details the association between BF% and incident hip fracture hospitalization stratified by sex. There was an inverse association between BF% and hip fracture amongst women. Compared to women with median BF% (39%), those with a lower BF% were at a significantly higher risk of incident hip fractures, up to 50% (1.52 (1.30-1.78)), while those with a higher BF% were at up to 25% lower risk of incident hip fractures (0.75 (0.58-0.97))). Compared to men with median BF% (23%), those with a lower BF% were at no statistically different risk of incident hip fractures, whilst those with a higher BF% were at a up to 49% higher risk of incident hip fractures (hazard ratio (95% confidence interval) = 1.49 (1.06-2.12)).

### Body fat percentage and broadband ultrasound attenuation

**Figure 2:**
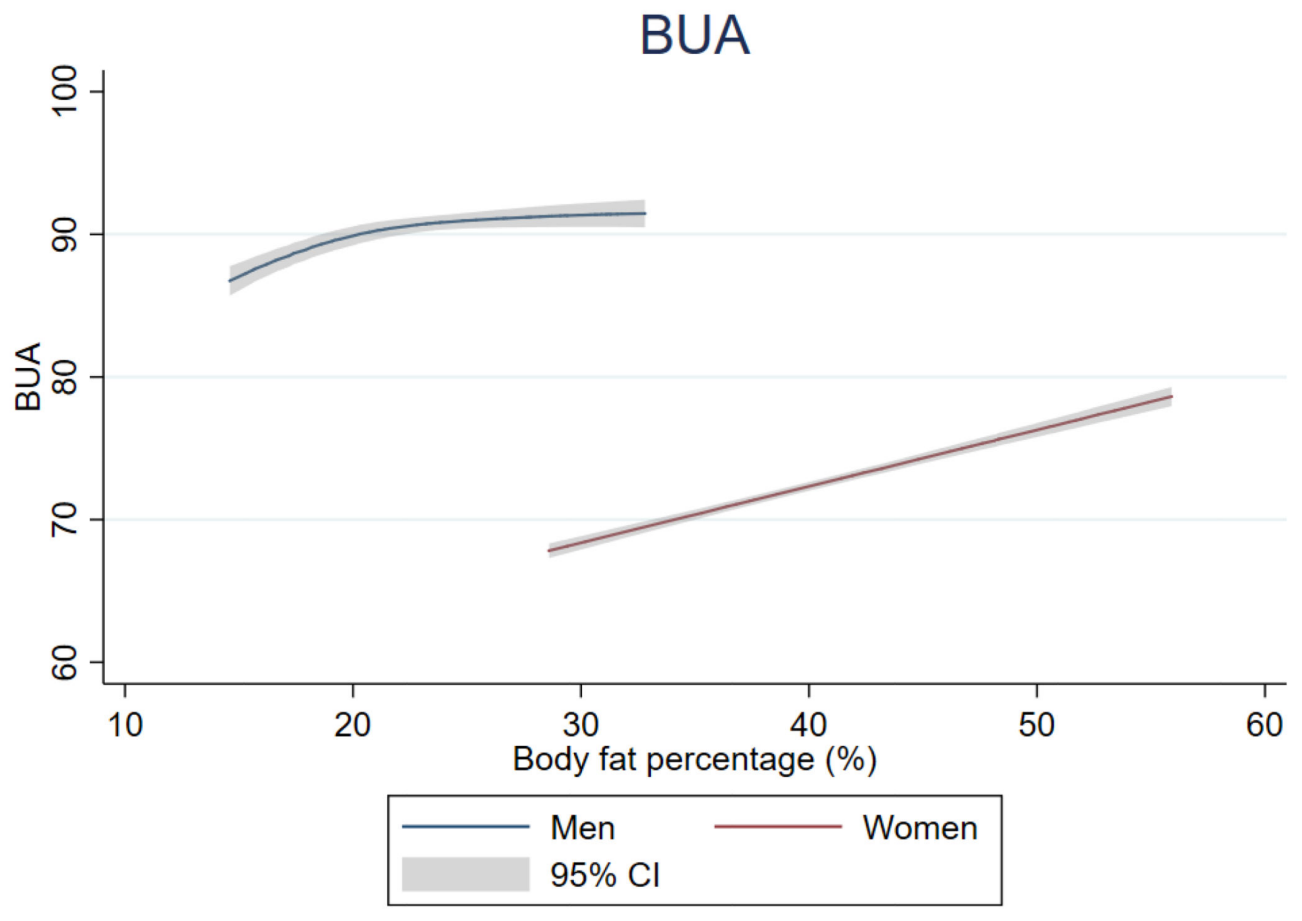
Results of multivariable restricted cubic spline (2 internal knots) and linear regression assessing the association between body fat percentage and broadband ultrasound attenuation in 14,129 included men and women participants of the EPIC-Norfolk study, respectively. The solid line denotes the point estimates of the broadband ultrasound attenuation as a function of body fat percentage. The grey area represents the bounds of the 95% confidence intervals. All models were adjusted for age, past history of fracture, height, smoking status and alcohol intake. BUA = broadband ultrasound attenuation (dB MHz^-1^); CI – confidence interval;

Figure 2 details the relationship between BF% and BUA, stratified by sex. Higher BF% was linearly associated with higher BUA amongst women (BUA increase for every 1% increase in BF% 0.40 (0.36-0.43)). In men, BUA ranging between 86.7 and 90.8 was associated with higher BF% between 14.6% and 23.3%. BF% values greater than 23% were associated with relatively constant BUA ~91.

### Stratified Analyses

**Figure 3:**
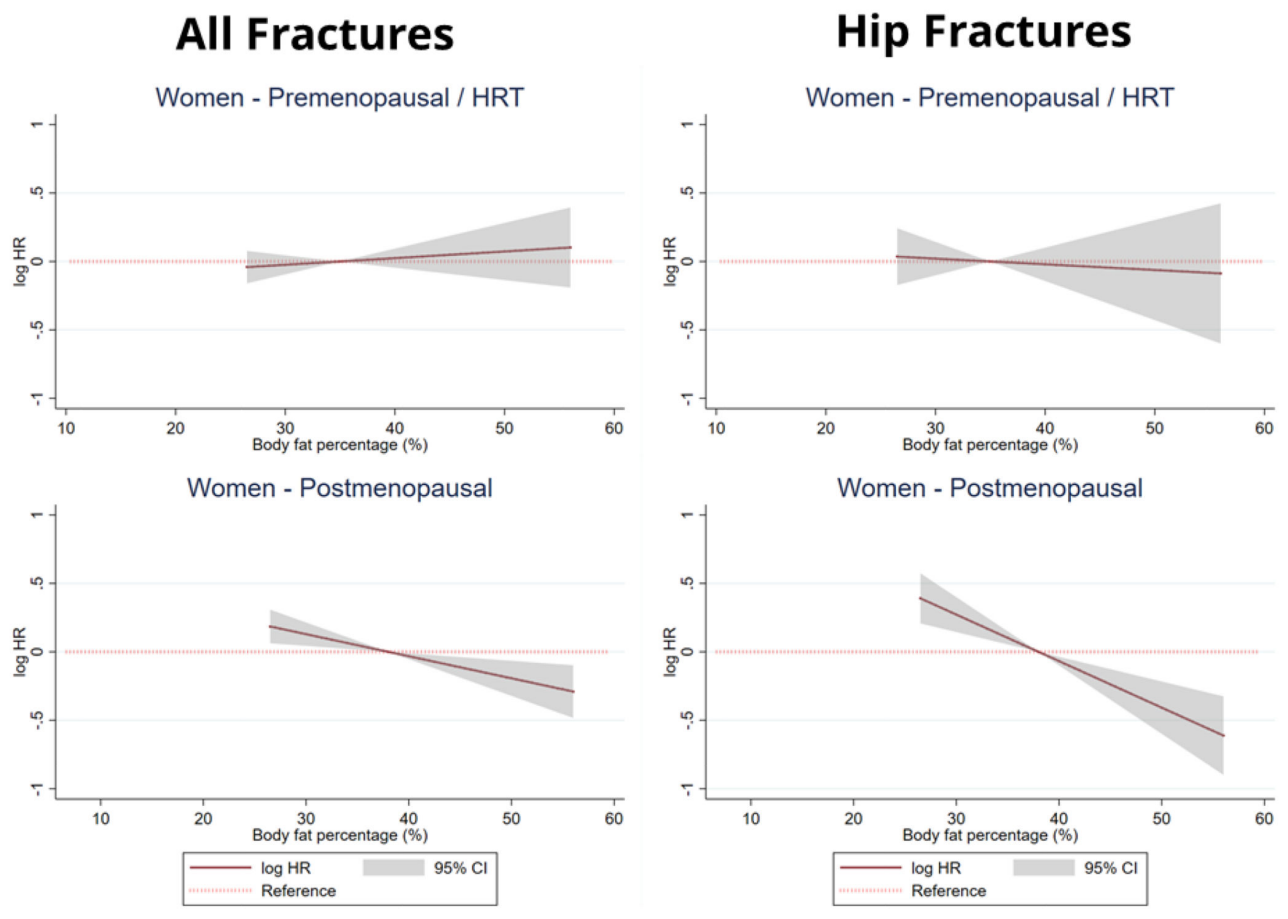
Results of multivariable Cox regressions assessing the association between body fat percentage (BF%) and incident all and hip fractures in 7946 included women participating in the EPIC-Norfolk, stratifying by hormonal milieu (pre-menopausal women and those undergoing HRT versus post-menopausal women). The hazard ratios describing the relationship between BF% and incident all fractures were modelled using a linear model for all subgroups. The natural logarithm of the hazard ratios and respective 95% confidence intervals are represented by the solid line and grey shadowing respectively. The dotted red line represents the reference line (log HR = 0 / HR = 1). Predicted hazard ratio values are displayed for BF% values ranging between the 5^th^ and 95^th^ percentiles of the analysed population. All models were adjusted for age, past history of fracture, height, smoking status, alcohol intake and broadband ultrasound attenuation. HR = hazard ratio; CI – confidence interval;

Figure 3 details the results of the analysis between BF% and incident all and hip fractures in women, stratifying by hormonal milieu (pre-menopausal women and those undergoing HRT versus post-menopausal women). There were no associations between BF% and incident fractures amongst pre-menopausal women and those undergoing HRT. The relationships between higher BF% and lower risk of incident fractures highlighted in the main analyses appeared to be largely driven by post-menopausal women. Amongst post-menopausal women, a 10% increase in BF% was associated with a 15% (0.85 (0.76-0.95)) and 29% (0.71 (0.61-0.83)) decrease in the risk of incident all and hip fractures respectively.

Supplementary Figure 1 details the results of the analysis between BF% and incident hip fractures in men, stratifying for testosterone levels (low vs high). There were no statistically significant associations between BF% and incident hip fractures in either group.

## Discussion

In this prospective population-based study of EPIC-Norfolk, we found that the relationship between body fat percent (BF%) and fracture hospitalisation differs between sexes. In women, the risk of hip fractures hospitalisation was lower with higher BF%. Nevertheless, in men the risk of all fracture hospitalisation did not change with BF%, while higher BF% was associated with higher risk of hip fracture hospitalisation. In women, higher BF% was linearly associated with higher bone mineral density estimated using calcaneal ultrasound. In men, higher BF% was associated with higher BUA only between ~14-23%, with no further associations with BF% >23%.

We found further evidence of gender differences in the relationship between BF% and fracture hospitalisation as well as possible explanations for this relationship in terms of estimated bone mineral density. The non-linear relationship between BF% and hospitalisation with all fractures among women, and the decreased risk of hip fracture hospitalisation with higher BF% is in concordance with the increased estimated bone mineral density (BMD) with higher BF% ^14^. The positive relationship between BMD and higher BF% may be attributed to increasing body weight inducing greater mechanical loading on bone ^6^. Furthermore, adipose tissue may be an important source of oestrogen in women, suppressing osteoclast activity with a resultant BMD increase ^7^. Fractures occur mostly due to falls, with the fracture risk influenced not only by bone mineral density but also by the mechanical force of the fall and the potential protective effect of surrounding soft tissue and the impact surface ^15–17^. The initial risk increase at lower BF% may be attributed to those with extremely low BF% being frailer and more susceptible to falls. In women with higher BF%, however, the reduction in hip fractures observed with BF% increase may be attributed to the potential cushioning effect of increased accumulation of adipose tissue within the gluteal region, which is further substantiated by the lack of reduction of hospitalization in any fracture with higher BF%, as excess adiposity may offer less protection for other bones

On the other hand, emerging evidence suggests that higher BF% may contribute to increased inflammation ^18^. This may lead to increased bone loss and osteopenia ^19,20^, which may explain the increased risk of hip fracture among men with higher BF%. Overall, these findings suggest that the effect of fat mass on bone may depend on bone type. In major weight bearing bones such at the femur, the increased body weight associated with higher BF% may mitigate the catabolic effect of inflammation ^7,21^ while in other bones inflammation may dominate. ^15–17,21^. Men, have lower BF% overall. Therefore, the potential cushioning effect against hip fractures described in women may not be apparent until the higher ranges of BF% of 24% for men. It is also possible that falls increase in men with higher BF% up to a threshold beyond which falls risk no longer increases as it is offset by extremely low physical activity. The increased falls risk in men with higher BF% may have occurred as a result of reduced muscle mass from the anti-androgenic effects of adiposity-related inflammation ^22,23^.

The results of our study also highlight the potential importance of hormonal milieu in mediating the associations between body fat and incident fractures. Amongst women, our stratified analyses revealed that the overall associations between higher BF% and incident fractures are driven mainly by postmenopausal women not undergoing HRT. There were no associations between BF% and incident fractures amongst women who were either premenopausal or were undergoing HRT, consistent with the protective role of female sex hormones against incident fractures. Our analyses assessing the effect of testosterone on the associations between body fat and incident hip fractures amongst men were likely underpowered to yield any conclusive results, given the high percentage of missing testosterone data in the EPIC-Norfolk study. Further research in this important question is therefore warranted.

We acknowledge some limitations. As a prospective population-based study, healthy responder bias and residual confounding need to be considered. Furthermore, BF% and bone mineral density were estimated using indirect methods of BIA and calcaneal BUA respectively, which are inferior to the gold standards of magnetic resonance imaging and dual-X-ray absorptiometry, which may influence the interpretation of the results. However, in large population studies magnetic resonance imaging and dual-X-ray absorptiometry are impractical and costly. In addition, BIA and BUA measurements were only obtained at one time-point, not accounting for potential changes in body composition over time. Future studies should consider incorporating serial measures benchmarked against gold standard measures. Furthermore, investigations should assess the independent relationship between BF% and fracture hospitalization after adjusting for bone mineral density and consider holistic strategies for fracture prevention taking into account falls risks, environmental hazards, physiological, physical and clinical attributes and other potential novel modifiable factors. As our fracture data was only ascertained after a hospital admission resulting from a fall, fractures that did not require hospitalisation would not have been captured in this study.

This study offers information obtained with a minimum of 15 years’ follow-up for a cohort of individuals with a mean age of 60 years at baseline. Having conducted a further analysis after a longer follow-up period since previously published study ^5^, we have been able to further determine the relationship between BF% and hip fracture hospitalization in addition to hospitalization from all fractures.

## Conclusion

The study findings add to the important evidence base regarding risk of a serious and prevalent health outcome. We found that over a 16-year follow-up period the risk of hip fractures reduces with higher BF% among women but increases with higher BF% in men with BF% greater than 23.0%. We therefore highlight for the first time the deleterious effects of higher BF% on the long-term incidence of hip fractures in men, which appears to occur independently of estimated bone mineral density. We therefore propose that future fracture prevention efforts also need to consider wider physical, clinical, physiological and environmental factors.

## Supplementary Material

Supplementary Materials

## Figures and Tables

**Table 1:** Baseline characteristics and crude outcome rates of the 7946 women included in the EPIC-Norfolk study stratified by quartiles of body fat percentage.

	WOMEN (N = 7946)				
	Body fat percentage				
	Quartile 1 <34.0%	Quartile 2 34.0-39.0%	Quartile 3 39.0-45.0%	Quartile 4 >45.0%	
N	2114	2018	1885	1929	
Age (years), mean (SD)	60.57 (9.76)	61.18 (9.00)	62.07 (8.65)	62.08 (8.28)	<0.001
Height (cm), mean (SD)	161.91 (6.24)	161.37 (6.11)	160.59 (6.02)	159.88 (5.96)	<0.001
Weight (kg), mean (SD)	58.58 (6.54)	65.45 (7.00)	70.99 (8.16)	80.67 (11.55)	<0.001
BMI (kg m^-2^), mean (SD)	22.33 (1.98)	25.11 (2.00)	27.49 (2.46)	31.53 (3.97)	<0.001
Waist-hip-ratio, mean (SD)	0.75 (0.05)	0.78 (0.06)	0.80 (0.06)	0.82 (0.06)	<0.001
VOS (m s^-1^), mean (SD)	1621.47 (43.61)	1624.59 (40.38)	1625.23 (38.68)	1629.00 (36.81)	<0.001
BUA (dB MHz^-1^), mean (SD)	68.74 (17.09)	71.58 (16.29)	73.01 (15.28)	75.90 (16.00)	<0.001
Age of menopause (years), mean (SD)	49.79 (5.07)	50.10 (5.41)	49.83 (5.55)	49.91 (5.26)	0.281
*Missing* (%)	201 (9.51%)	123 (6.10%)	78 (4.14%)	74 (3.84%)	
Alcohol intake (units per week), median (IQR)	2.50 (1.00-7.00)	2.50 (1.00-7.00)	2.00 (1.00-7.00)	2.00 (1.00-5.50)	<0.001
Smoking status, N (%)					<0.001
Current smoker	218 (10.31%)	166 (8.23%)	126 (6.68%)	136 (7.05%)	
Former smoker	607 (28.71%)	613 (30.38%)	625 (33.16%)	724 (37.53%)	
Never smoker	1289 (60.97%)	1239 (61.40%)	1134 (60.16%)	1069 (55.42%)	
Physical activity levels, N (%)					<0.001
Inactive	145 (6.86%)	127 (6.29%)	142 (7.53%)	215 (11.15%)	
Moderately inactive	791 (37.42%)	814 (40.34%)	788 (41.80%)	827 (42.87%)	
Moderately active	569 (26.92%)	483 (23.93%)	451 (23.93%)	452 (23.43%)	
Active	609 (28.81%)	594 (29.44%)	504 (26.74%)	435 (22.55%)	
Menopausal status at the 2^nd^ health check, N (%)					<0.001
Pre-menopausal	189 (8.94%)	111 (5.50%)	72 (3.82%)	71 (3.68%)	
Peri-menopausal	82 (3.88%)	72 (3.57%)	60 (3.18%)	47 (2.44%)	
Post-menopausal (1-5 years after last period)	415 (19.63%)	359 (17.79%)	317 (16.82%)	310 (16.07%)	
Post-menopausal (>5 years after last period)	1369 (64.76%)	1401 (69.43%)	1376 (73.00%)	1445 (74.91%)	
*Missing* (%)	59 (2.79%)	75 (3.72%)	60 (3.18%)	56 (2.90%)	
HRT use at the 2^nd^ health check, N (%)					<0.001
Current	487 (23.04%)	454 (22.50%)	376 (19.95%)	369 (19.13%)	
Former	326 (15.42%)	359 (17.79%)	335 (17.77%)	405 (21.00%)	
Never	1301 (61.54%)	1204 (59.66%)	1173 (62.23%)	1154 (59.82%)	
Reasons for HRT prescription					0.001
Menopausal symptoms	438 (20.72%)	474 (23.49%)	409 (21.70%)	456 (23.64%)	
Osteoporosis	83 (3.93%)	53 (2.63%)	57 (3.02%)	37 (1.92%)	
Other reasons	162 (7.66%)	132 (6.54%)	128 (6.79%)	146 (7.57%)	
*Missing* (%)	1431 (67.69%)	1359 (67.34%)	1291 (68.49%)	1290 (66.87%)	
Past history of fracture	177 (8.37%)	156 (7.73%)	135 (7.16%)	159 (8.24%)	0.486
All incident fractures	267 (12.63%)	241 (11.94%)	199 (10.56%)	202 (10.47%)	0.087
Incident hip fractures	137 (6.48%)	113 (5.60%)	76 (4.03%)	73 (3.78%)	<0.001

SD=standard deviation, IQR=inter-quartile range, BMI=body mass index; VOS=velocity of sound; BUA= broadband ultrasound attenuation

*P*-values for between-group differences were derived using one-way analysis of variance (normally-distributed continuous data), the Kruskal-Wallis test (non-normally distributed continuous data) or thechi-squared test (categorical data).

**Table 2:** Baseline characteristics and crude outcome rates of the 6183 men included in the EPIC-Norfolk study stratified by quartiles of body fat percentage.

	MEN (N = 6183)				
	Body fat percentage				
	Quartile 1 <19.8%	Quartile 2 19.8-23.0%	Quartile 3 23.0-27.2.0%	Quartile 4 >27.2%	
N	1643	1506	1533	1492	
Age (years), mean (SD)	63.10 (9.44)	62.86 (8.90)	62.80 (8.77)	62.84 (8.64)	0.776
Height (cm), mean (SD)	174.43 (6.89)	173.90 (6.47)	173.95 (6.38)	173.55 (6.52)	0.002
Weight (kg), mean (SD)	71.51 (7.51)	78.56 (7.15)	83.92 (8.06)	92.43 (10.94)	<0.001
BMI (kg m^-2^), mean (SD)	23.48 (1.79)	25.95 (1.48)	27.69 (1.68)	30.65 (2.82)	<0.001
Waist-hip-ratio, mean (SD)	0.88 (0.05)	0.92 (0.05)	0.94 (0.05)	0.97 (0.05)	<0.001
VOS (m s^-1^), mean (SD)	1648.22 (41.90)	1647.46 (40.23)	1645.12 (38.79)	1640.63 (37.67)	<0.001
BUA (dB MHz^-1^), mean (SD)	88.34 (18.54)	90.01 (17.52)	91.30 (16.95)	90.83 (16.75)	<0.001
Testosterone levels (nmol/L), mean (SD)	17.83 (5.82)	16.70 (5.81)	16.50 (5.56)	15.78 (5.44)	<0.001
*Missing* (%)	958 (58.17%)	937 (62.18%)	936 (61.02%)	901 (60.27%)	
Alcohol intake (units per week), median (IQR)	6.00 (2.00-12.50)	6.00 (2.00-14.00)	7.00 (2.00-14.50)	7.00 (2.00-16.00)	0.001
Smoking status, N (%)					<0.001
Current smoker	168 (10.20%)	114 (7.56%)	104 (6.78%)	110 (7.36%)	
Former smoker	772 (46.87%)	805 (53.42%)	918 (59.84%)	956 (63.95%)	
Never smoker	707 (42.93%)	588 (39.02%)	512 (33.38%)	429 (28.70%)	
Physical activity levels, N (%)					<0.001
Inactive	109 (6.62%)	82 (5.44%)	114 (7.43%)	155 (10.37%)	
Moderately inactive	547 (33.21%)	459 (30.46%)	456 (29.73%)	508 (33.98%)	
Moderately active	354 (21.49%)	342 (22.69%)	354 (23.08%)	286 (19.13%)	
Active	637 (38.68%)	624 (41.41%)	610 (39.77%)	546 (36.52%)	
Past history of fracture	95 (5.77%)	90 (5.97%)	86 (5.61%)	102 (6.82%)	0.503
All incident fractures	96 (5.83%)	89 (5.91%)	93 (6.06%)	96 (6.42%)	0.905
Incident hip fractures	41 (2.49%)	22 (1.46%)	33 (2.15%)	42 (2.81%)	0.074

SD=standard deviation, IQR=inter-quartile range, BMI=body mass index; VOS=velocity of sound; BUA= broadband ultrasound attenuation

*P*-values for between-group differences were derived using one-way analysis of variance (normally-distributed continuous data), the Kruskal-Wallis test (non-normally distributed continuous data) or thechi-squared test (categorical data).
