# Supplementary material for: Body Fat Percentage and Long-Term Risk of Fractures. The EPIC-Norfolk Prospective Population Cohort Study
Source: Maturitas. Author manuscript; Available in PMC 2023 May 23. (PMC7614563; doi:10.1016/j.maturitas.2022.11.005)
Supplement: Supplementary Materials [file EMS175904-supplement-Supplementary_Materials.docx]

**Supplementary Data**

**Supplementary Table 1**. Best fitting models chosen for each Cox regression analysis.

| **Main analyses** | | | |
| --- | --- | --- | --- |
|  | All incident fractures | Incident hip fractures | BUA |
| All women | RCS – df = 3 | RCS – df = 2 | Linear |
| All men | RCS – df = 3 | RCS – df = 3 | RCS – df = 3 |
| **Other analyses** | | | |
| Women (premenopausal/HRT) | Linear | Linear | - |
| Women (postmenopausal) | Linear | Linear |  |
| Men  (low testosterone) | - | Linear |  |
| Men  (normal testosterone) | - | RCS – df = 3 |  |

BUA - broadband ultrasound attenuation; WHR – waist-hip ratio; RCS – restricted cubic splines; df – degrees of freedom.

**Supplementary Table 2.** International Classification of Disease, Tenth Edition (ICD-10) codes used to classify fracture diagnoses as well as the number of incident fractures recorded during the study follow-up, stratified by sex.

|  | Women (N = 7946) | Men (N = 6183) |
| --- | --- | --- |
| All fractures  (ICD-10 S02, S12, S22, S32, S42, S52, S62, S72, S82, S92) | 908 | 374 |
| Hip fractures  (ICD-10 S72) | 399 | 138 |
| Wrist fractures  (ICD-10 S62) | 245 | 71 |

ICD-10 - International Classification of Disease, Tenth Edition

**Supplementary Table 3.** Baseline characteristics and crude outcome rates of the 7946 **women** included in the EPIC-Norfolk study stratified by the median of body fat percentage.

|  | **Total** | **BF% <=39.0** | **BF% >39.0** | *P*-value |
| --- | --- | --- | --- | --- |
| N | 7946 | 4132 | 3814 |  |
| Age (years),  mean (SD) | 61.45 (8.98) | 60.87 (9.40) | 62.08 (8.46) | <0.001 |
| Height (cm),  mean (SD) | 160.97 (6.13) | 161.65 (6.18) | 160.23 (6.00) | <0.001 |
| Weight (kg),  mean (SD) | 68.63 (11.74) | 61.93 (7.59) | 75.89 (11.13) | <0.001 |
| BMI (kg m^-2^),  mean (SD) | 26.49 (4.34) | 23.68 (2.43) | 29.53 (3.88) | <0.001 |
| Waist-hip-ratio,  mean (SD) | 0.79 (0.06) | 0.77 (0.05) | 0.81 (0.06) | <0.001 |
| VOS (m s^-1^),  mean (SD) | 1624.98 (40.13) | 1623.00 (42.09) | 1627.13 (37.79) | <0.001 |
| BUA (dB MHz^-1^),  mean (SD) | 72.21 (16.41) | 70.13 (16.76) | 74.47 (15.71) | <0.001 |
| Age of menopause (years), mean (SD) | 49.91 (5.32) | 49.95 (5.24) | 49.87 (5.40) | 0.530 |
| *Missing* (%) | 476 (5.99%) | 324 (7.84%) | 152 (3.99%) |  |
| Alcohol intake (units per week),  median (IQR) | 2.00  (1.00-7.00) | 2.50  (1.00-7.00) | 2.00  (1.00-6.00) | <0.001 |
| Smoking status,  N (%) |  |  |  | <0.001 |
| Current smoker | 646 (8.13%) | 384 (9.29%) | 262 (6.87%) |  |
| Former smoker | 2569 (32.33%) | 1220 (29.53%) | 1349 (35.37%) |  |
| Never smoker | 4731 (59.54%) | 2528 (61.18%) | 2203 (57.76%) |  |
| Physical activity levels, N (%) |  |  |  | <0.001 |
| Inactive | 629 (7.92%) | 272 (6.58%) | 357 (9.36%) |  |
| Moderately inactive | 3220 (40.52%) | 1605 (38.84%) | 1615 (42.34%) |  |
| Moderately active | 1955 (24.60%) | 1052 (25.46%) | 903 (23.68%) |  |
| Active | 2142 (26.96%) | 1203 (29.11%) | 939 (24.62%) |  |
| Menopausal status at the 2^nd^ health check, N (%) |  |  |  | <0.001 |
| Pre-menopausal | 443 (5.58%) | 300 (7.26%) | 143 (3.75%) |  |
| Peri-menopausal | 261 (3.28%) | 154 (3.73%) | 107 (2.81%) |  |
| Post-menopausal (1-5 years after last period) | 1401 (17.63%) | 774 (18.73%) | 627 (16.44%) |  |
| Post-menopausal (>5 years after last period) | 5591 (70.36%) | 2770 (67.04%) | 2821 (73.96%) |  |
| *Missing (%)* | 250 (3.15%) | 134 (3.24%) | 116 (3.04%) |  |
| HRT use at the 2^nd^ health check, N (%) |  |  |  | <0.001 |
| Current | 1686 (21.22%) | 941 (22.77%) | 745 (19.53%) |  |
| Former | 1425 (17.93%) | 685 (16.58%) | 740 (19.40%) |  |
| Never | 4832 (60.81%) | 2505 (60.62%) | 2327 (61.01%) |  |
| Reasons for HRT prescription |  |  |  | 0.082 |
| Menopausal symptoms | 1777 (22.36%) | 912 (22.07%) | 865 (22.68%) |  |
| Osteoporosis | 230 (2.89%) | 136 (3.29%) | 94 (2.46%) |  |
| Other reasons | 568 (7.15%) | 294 (7.12%) | 274 (7.18%) |  |
| *Missing (%)* | 5371 (67.59%) | 2790 (67.52%) | 2581 (67.67%) |  |
| Past history of fracture | 627 (7.89%) | 333 (8.06%) | 294 (7.71%) | 0.562 |
| All incident fractures | 909 (11.44%) | 508 (12.29%) | 401 (10.51%) | 0.013 |
| Incident hip fractures | 399 (5.02%) | 250 (6.05%) | 149 (3.91%) | <0.001 |

SD=standard deviation, IQR=inter-quartile range, BMI=body mass index; VOS=velocity of sound; BUA= broadband ultrasound attenuation

*P*-values for between-group differences were derived using one-way analysis of variance (normally-distributed continuous data), the Kruskal-Wallis test (non-normally distributed continuous data) or thechi-squared test (categorical data) .

**Supplementary Table 4.** Baseline characteristics and crude outcome rates of the 6183 **men** included in the EPIC-Norfolk study stratified by the median of body fat percentage.

|  | **Total** | **BF% <=23.0** | **BF% >23.0** | *P*-value |
| --- | --- | --- | --- | --- |
| N | 6183 | 3154 | 3029 |  |
| Age (years),  mean (SD) | 62.90 (8.95) | 62.98 (9.19) | 62.82 (8.71) | 0.469 |
| Height (cm),  mean (SD) | 173.97 (6.58) | 174.18 (6.70) | 173.75 (6.45) | 0.011 |
| Weight (kg),  mean (SD) | 81.37 (11.47) | 74.88 (8.14) | 88.12 (10.49) | <0.001 |
| BMI (kg m^-2^),  mean (SD) | 26.86 (3.30) | 24.66 (2.06) | 29.15 (2.75) | <0.001 |
| Waist-hip-ratio,  mean (SD) | 0.93 (0.06) | 0.90 (0.05) | 0.95 (0.05) | <0.001 |
| VOS (m s^-1^),  mean (SD) | 1645.43 (39.83) | 1647.86 (41.10) | 1642.90 (38.30) | <0.001 |
| BUA (dB MHz^-1^),  mean (SD) | 90.08 (17.51) | 89.14 (18.08) | 91.07 (16.85) | <0.001 |
| Testosterone levels (nmol/L), mean (SD) | 16.74 (5.71) | 17.32 (5.84) | 16.14 (5.51) | <0.001 |
| *Missing* (%) | 3732 (60.36%) | 1895 (60.08%) | 1837 (60.65%) |  |
| Alcohol intake (units per week),  median (IQR) | 6.50  (2.00-14.00) | 6.00  (2.00-13.00) | 7.00  (2.00-15.00) | <0.001 |
| Smoking status,  N (%) |  |  |  | <0.001 |
| Current smoker | 496 (8.02%) | 282 (8.94%) | 214 (7.07%) |  |
| Former smoker | 3451 (55.81%) | 1577 (50.00%) | 1874 (61.87%) |  |
| Never smoker | 2236 (36.16%) | 1295 (41.06%) | 941 (31.07%) |  |
| Physical activity levels, N (%) |  |  |  | <0.001 |
| Inactive |  |  |  |  |
| Moderately inactive | 460 (7.44%) | 191 (6.06%) | 269 (8.88%) |  |
| Moderately active | 1970 (31.86%) | 1006 (31.90%) | 964 (31.83%) |  |
| Active | 1336 (21.61%) | 696 (22.07%) | 640 (21.13%) |  |
| Past history of fracture | 373 (6.03%) | 185 (5.87%) | 188 (6.21%) | 0.573 |
| All incident fractures | 374 (6.05%) | 185 (5.87%) | 189 (6.24%) | 0.537 |
| Incident hip fractures | 138 (2.23%) | 63 (2.00%) | 75 (2.48%) | 0.203 |

SD=standard deviation, IQR=inter-quartile range, BMI=body mass index; VOS=velocity of sound; BUA= broadband ultrasound attenuation

*P*-values for between-group differences were derived using one-way analysis of variance (normally-distributed continuous data), the Kruskal-Wallis test (non-normally distributed continuous data) or thechi-squared test (categorical data).


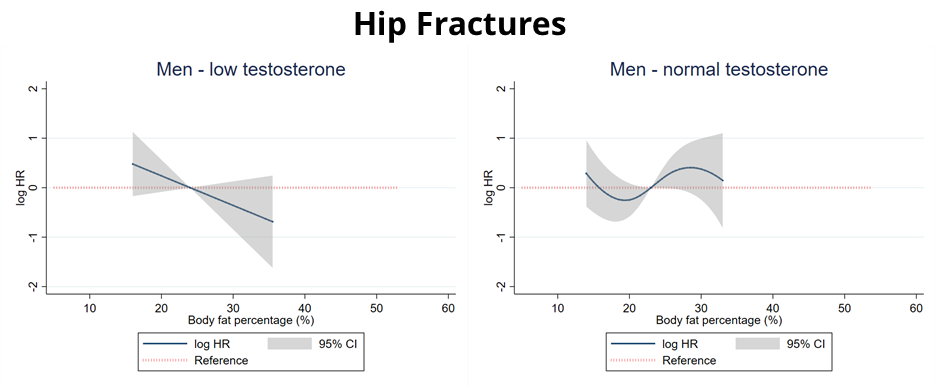
**Supplementary Figure 1.** Results of multivariable Cox regressions assessing the association between body fat percentage (BF%) and incident hip fractures in 2447 included men with available testosterone data from the EPIC-Norfolk study over the follow-up period, stratifying by testosterone levels (low <25^th^ percentile vs normal levels). The hazard ratios describing the relationship between BF% and incident all fractures were modelled using a linear model for men with low testosterone levels and a restricted cubic spline model with 2 internal knots for men with normal testosterone levels. The natural logarithm of the hazard ratios and respective 95% confidence intervals are represented by the solid line and grey shadowing respectively. The dotted red line represents the reference line (log HR = 0 / HR = 1). Predicted hazard ratio values are displayed for BF% values ranging between the 5^th^ and 95^th^ percentiles of the analysed population.

All models were adjusted for age, past history of fracture, height, smoking status, alcohol intake and broadband ultrasound attenuation.

HR = hazard ratio; CI – confidence interval;
